# A new microscopic method to analyse desiccation‐induced volume changes in aeroterrestrial green algae

**DOI:** 10.1111/jmi.12409

**Published:** 2016-04-14

**Authors:** K. LAJOS, S. MAYR, O. BUCHNER, K. BLAAS, A. HOLZINGER

**Affiliations:** ^1^ University of Innsbruck, Institute of BotanyFunctional Plant BiologySternwartestrasse 156020InnsbruckAustria; ^2^ Present address: Szent István UniversityPlant Protection InstitutePáter Károly utca 1H‐2100GödöllőHungary

**Keywords:** Desiccation, hydraulic parameter, light microscopy, Streptophyta, water loss

## Abstract

Aeroterrestrial green algae are exposed to desiccation in their natural habitat, but their actual volume changes have not been investigated. Here, we measure the relative volume reduction (RV_RED_) in *Klebsormidium crenulatum* and *Zygnema* sp. under different preset relative air humidities (RH). A new chamber allows monitoring RH during light microscopic observation of the desiccation process. The RHs were set in the range of ∼4 % to ∼95% in 10 steps. RV_RED_ caused by the desiccation process was determined after full acclimation to the respective RHs. In *K. crenulatum*, RV_RED_ (mean ± SE) was 46.4 ± 1.9%, in *Zygnema* sp. RV_RED_ was only 34.3 ± 2.4% at the highest RH (∼95%) tested. This indicates a more pronounced water loss at higher RHs in *K. crenulatum* versus *Zygnema* sp. By contrast, at the lowest RH (∼4%) tested, RV_RED_ ranged from 75.9 ± 2.7% in *K. crenulatum* to 83.9 ± 2.2% in *Zygnema* sp. The final volume reduction is therefore more drastic in *Zygnema* sp. These data contribute to our understanding of the desiccation process in streptophytic green algae, which are considered the closest ancestors of land plants.

## Lay description

Green algae living on the surface of soil are potentially exposed to drying. When they lose water, the volume of the alga is changed, but currently no measurements of these changes are available. We here investigate two green algae from different classes. The algae are exposed to defined relative air humidities in a specially constructed microscopic chamber, and the volume changes are measured. A distinct behaviour of the two investigated algae gives new insights in the lifestyle of terrestrial algae, the closest relatives of land plants.

## Introduction

Green algae have the ability to perform photosynthesis and their major distribution is in aquatic habitats making them rather an ecological unit than a taxonomic entity. In fact, according to recent phylogenetic research, green algae can be divided in two main lineages (Leliaert *et al*., 2012): (1) Chlorophyta that contains the majority of described green algal species and (2) Streptophyta that includes the Charophytes, a paraphyletic group of freshwater and terrestrial green algae, as well as all land plants, the Embryophytes (Leliaert *et al*., 2012). Among both lineages, several groups with terrestrial representatives occur. In case of the Chlorophyta, the classes Ulvophyceae, Trebouxiophyceae and Chlorophyceae include some taxa living in terrestrial habitats, e.g. the Trentepohliales of the Ulvophyceae (Leliaert *et al*., 2012), the members of Trebouxiophyceae (‘lichen algae group’) and sparse genera in the Chlorophyceae, e.g. *Fritschiella* (Holzinger & Karsten, 2013). In the streptophyte lineage, terrestrial members can be found in the classes Klebsormidiophyceae, Zygnematophyceae and Coleochaetopyhceae (Graham *et al*., 2012; Holzinger & Karsten, 2013; Herburger & Holzinger, 2015; Mikhailyuk *et al*., 2015). The closest relatives to land plants can be found among charophyte green algae (Wodniok *et al*., 2011; Timme *et al*., 2012; Delwiche & Cooper, 2015). Their genome holds primary factors for plant terrestrial adaptation (Hori *et al*., 2014), and transcriptional changes upon severe desiccation stress demonstrated a land plant like defence reaction in *Klebsormidium* (Holzinger *et al*., 2014). Raffinose family oligosaccharides have been found to be upregulated upon desiccation stress likely contributing to the osmotic potential (Holzinger *et al*., 2014). Increasing knowledge on cell wall properties of these algal groups has become available recently, demonstrating the close relationship to land plants (Sørensen *et al*., 2011; Mikhailyuk *et al*., 2014; Herburger & Holzinger, 2015). However, green algae are poikilhydric organisms that do not have cuticles or similar structures to protect from water evaporation (Delaux *et al*., 2013).

Although the hydraulic parameters (osmotic potential at full turgor and relative water content at the turgor loss point) in pokilohydric plants of the embryophytes, ferns, mosses and lichens have been well investigated (see Table 1), only little information is available in aeroterrestrial green algae (Holzinger & Karsten, 2013). This lack of data may be related to the difficulty of determining the volume or weight of terrestrial green algal samples due to their small size, mostly ranging between 5 and 25 μm in diameter (Rindi *et al*., 2011; Herburger *et al*., 2015). By contrast, the osmotic potential has been determined in *Klebsormidium* and *Zygnema* (Kaplan *et al*., 2012, 2013). In these studies, the water potential at the turgor loss point (Ψ_TLP_) was determined by means of the ‘incipient plasmolysis technique’. For plasmolysis fully turgescent plant cells, like the cell filaments of the investigated green algae, were exposed to osmotically active solutions with increasing concentrations and the value where 50% of the cells plasmolyzed determined microscopically. At the equilibrium point, the cells have the same osmolarity as the external solution, which allowed determining the osmotic potential. In *K. crenulatum* a Ψ_TLP_ of ‐2.09 MPa whereas in *Klebsormidium nitens* a Ψ_TLP_ of ‐1.67 MPa was found (Kaplan *et al*., 2012). In the same way, Ψ_TLP_ values of ‐1.67 and ‐0.8 MPa were determined in different strains of *Zygnema* sp. (Kaplan *et al*., 2013).

**Table 1:** Osmotic potential at full turgor (Ψ_osat_) and relative water content at the turgor loss point = incipient plasmolysis (RWC_TVP_) of different poikilohydric species of angiosperms, ferns, mosses and lichens. The references, from which the data were obtained, are also listed

Species name	Ψ_osat_ (‐MPa)	RWC_TVP_ (%)	References
**Angiosperms**			
Dicotyledons			
*Myrothamnus flabellifolia*	1.92	90	Beckett (1997)
Monocotyledons			
*Xerophyta scabrida*	0.55	―	Tuba *et al*. (1994)
*Xerophyta viscosa*	1.41	81	Beckett (1997)
**Ferns**			
*Gymnopteris hispida*	1.3	―	Walter & Stadelmann (1974)
*Cheilanthes lindheimeri*	2.4	―	―‖―
*Trichomanes melanotrichum*	1.77	78	Beckett (1997)
**Mosses**			
*Dumortiera hirsuta*	0.38	85	Proctor *et al*. (1998)
*Marchantia polymorpha*	0.38	60	―‖―
*Conocephalum conicum*	0.54	45	―‖―
*Dumortiera hirsuta*	0.59	94	Beckett (1997)
*Hookeria lucens*	0.95	70	Proctor *et al*. (1998)
*Sphagnum girgensohnii*	0.98	36	Hájek & Beckett (2008)
*Atrichum androgynum*	1.05	77	―‖―
*Sphagnum cuspidatum*	1.08	62	―‖―
*Sphagnum tenellum*	1.09	41	―‖―
*Sphagnum magellanicum*	1.12	61	―‖―
*Mnium hornum*	1.21	70	Proctor *et al*. (1998)
*Neckera crispa*	1.27	65	―‖―
*Sphagnum fuscum*	1.31	61	Hájek & Beckett (2008)
*Rhytidiadelphus loreus*	1.34	70	Proctor *et al*. (1998)
*Tortula ruralis*	1.36	75	―‖―
*Antitrichia curtipendula*	1.47	65	―‖―
*Andreaea alpina*	1.59	70	―‖―
*Anomodon viticulosus*	1.65	65	―‖―
*Frullania tamarisci*	1.78	60	―‖―
*Porella capensis*	1.89	71	Beckett (1997)
*Homalothecium lutescens*	2.08	70	Proctor *et al*. (1998)
*Polytrichum commune*	2.09	75	―‖―
*Plagiomnium rhynchophorum*	2.37	55	Beckett (1997)
**Lichens**			
*Peltigera leucophlebia*	0.62	70	Nardini *et al*. (2013)
*Peltigera canina*	1.00	59	Beckett (1995)
*Lobaria scrobiculata*	1.43	54	―‖―
*Cladonia convoluta*	1.52	60	Proctor *et al*. (1998)
*Peltigera rufescens*	1.55	60	Nardini *et al*. (2013)
*Sticta limbata*	1.61	51	Beckett (1995)
*Ramalina celastri*	1.95	44	―‖―
*Roccella hypomecha*	2.19	42	Beckett (1997)
*Usnea undulata*	2.19	46	Beckett (1995)
*Pseudocyphellaria aurata*	2.23	49	―‖―
*Parmotrema tinctorum*	2.52	47	―‖―
*Roccella montagnei*	2.79	43	―‖―

In this study, we developed and tested a special chamber that allows microscopic observations at several preset relative air humidity (RH) levels. To illustrate the capacities of this chamber, we analysed the desiccation‐induced volume changes of the cytoplasm in streptophyte green algae. We hypothesized that green algae from distinct classes (Klebsormidiophyceae, Zygnematophycae) show different desiccation patterns. We measured the relative volume reduction (RV_RED_) of the protoplast at different preset RH levels under controlled conditions. This allowed us to get information on the water loss at different RHs and to enhance our understanding of desiccation processes in the studied organisms.

## Materials and methods

### Plant material

Two strains from different charophyte classes were selected for the experiments, as contrasting desiccation behaviour was expected from previous studies (Kaplan *et al*., 2012, 2013).

(1) *Klebsormidium crenulatum* (Kützing) Lokhorst (Lokhorst & Star, 1985) was previously isolated from an alpine soil sample (Obergurgl, Tyrol, Austria, 46° 50’ 99.8’’ N, 11° 00’ 90.3’’ E; 2,350 m a.s.l.; Karsten *et al*., 2010; Culture Collection of Algae Göttingen, SAG 2415). *K. crenulatum* was cultivated in liquid‐modified Bold's Basal Medium (BBM; Starr & Zeikus, 1993) at a light dark regime of 16–8 h at 20°C during light and 15°C during dark phase (Kaplan *et al*., 2012).

(2) *Zygnema* sp. (C.Agardh) ‘Saalach’ (SAG 2419), previously collected from the sandy littoral zone of the River Saalach near the City of Salzburg (Austria, 47°47′ 8.70″ N, 12°56′ 42.66″ O; 440 m a.s.l.; Herburger *et al*., 2015). *Zygnema* sp. was cultivated in conventional BBM (Bischoff & Bold, 1963) at the same light‐dark regime and temperature as described above.

### Test chamber

**Figure 1:**
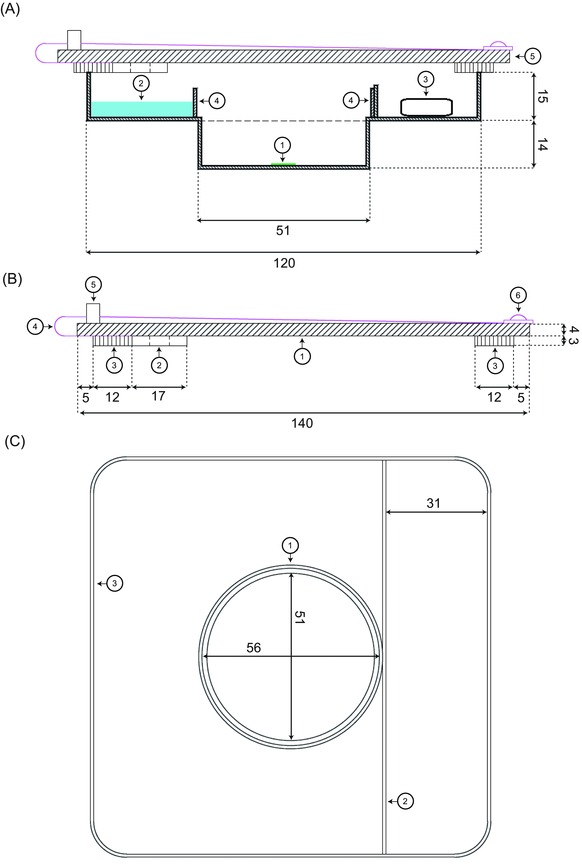
Two‐storied test chamber. (A) Two‐dimensional, cross‐sectional view of the test chamber during a desiccation experiment. Bottom storey with: (1) algal sample; top storey with (2) salt solution; (3) air humidity and temperature sensor; (4) plastic ring of 9 mm height, preventing the contact of the salt solution with the investigated specimen; (5) Perspex lid. (B) Detailed description of the Perspex lid: (1) square panel of Perspex, forming the central body of the lid; (2) cross‐section of one of the two motorized mini fans; (3) sponge rubber seal; (4) power supply cable of one of the two motorized mini fans; (5) power adapter; (6) screw fixing the power supply cable. (C) Top view of the test chamber: (1) plastic ring, (4) in (A); (2) linear plastic delimitation of 10 mm height, preventing a contact of the salt solution with the sensor; (3) outer margin of the petri dish. All dimensions in millimetre.

A test chamber that allowed: (1) maintaining a constant RH for desiccation experiments and (2) microscopic observation of the samples was constructed (Fig. 1). The test chamber was two‐storied, where the ‘upper storey’ consisted of a 120 × 120 mm square transparent polycarbonate Petri dish (Nr. 18252, Gammarad, Bologna, Italy). There, the salt solution or silica gel and in a separated space (created by a 10 mm plastic delimitation) the air humidity sensor (mini data logger PCE‐MSR145W‐THPA, PCE Instruments, Meschede, Germany) were placed. This ‘upper storey’ had a circular hole, where a smaller, circular Petri dish (60 mm Ø, Nr. 628160, Greiner Labortechnik, Kremsmünster, Austria) was attached as the ‘lower storey’ which fitted into the mechanical stage of the microscope. In this ‘lower storey’, the algal samples were directly placed for observation. To separate the sample from the salt solution or silica gel, a plastic ring (9 mm high, made from the lid of round Petri dish), was attached around the hole of the upper Petri dish. The test chamber was covered by a square lid with a rubber seal (140 × 140 mm, 4 mm thick, transparent acrylic glass). Two motorized mini fans (MagLev Motor Blower, Spec. No. D01000940G‐00, 17 × 17 × 3 mm, Sunonwealth Electric Machine Industry, Kaohsiung City, Taiwan) were attached to the lid, and ensured a constant ventilation of the chamber.

### Experimental setting of the RH

The precise setting of the RH inside the hermetically sealed test chamber was achieved by different nonsaturated lithium chloride (LiCl) solutions (≥98%, Sigma‐Aldrich, Vienna, Austria) according to Hay *et al*. (2008), see Table 2. Alternatively, a saturated K_2_CO_3_ solution (≥99.0%, Sigma‐Aldrich) was used. The lowest RH generated by saturated LiCl solution is 11.2% at 20°C (Hay *et al*., 2008). Therefore, silica gel (with moisture indicator, Sigma‐Aldrich) was used to generate RH values of 3.7–4.2% (Table 2).

**Table 2:** Salt solutions and silica gel used to generate different RH levels in the test chamber; slightly different values were measured after equilibration of the test organisms *Klebsormidium crenulatum* and *Zygnema* sp

Material	Quantity (g/100 mL)	RH (%) *K. crenulatum*	RH (%) *Zygnema* sp.
Lithium chloride (LiCl)	4.8	95.4	95.8
	9.4	89.5	91.7
	13.0	85.3	85.3
	17.1	78.3	78.4
	24.1	65.5	66.4
	30.0	56.9	56.4
	43.5	31.8	32.3
	64.1	15.9	15.6
Potassium carbonate (K_2_CO_3_)	112	48.1	49.5
Silica gel	―	3.7	4.2

### Experimental procedure

The test organisms (*K. crenulatum*, *Zygnema* sp.) were placed in a volume of 20 μL culture medium in the ‘lower storey’ of the test chamber. In the ‘upper storey’, the respective salt solution or silica gel was filled. The cell filaments were spread and microscopic images were taken immediately. The cells were then allowed to completely equilibrate for up to 12 h at 20°C to the respective RH before 10–15 random images were taken. The measurements of the diameters at individual protoplasts were performed only on filaments that were found solitary after desiccation, where the protoplasts as well as the cell lumen diameter (CLD) were clearly visible. In *K. crenulatum*, an average of 22 protoplasts per RH step and in *Zygnema* sp. an average of 16 protoplasts per RH step were measured.

### Determination of the RV_RED_

The desiccation process was monitored by an inverted Zeiss Axiovert 200M microscope (20x, NA = 0.50; 40x, NA = 0.75; Carl Zeiss AG, Oberkochen, Germany). To determine the protoplast volume, the CLD and the diameter of the desiccated protoplast (PD_d_) were measured in individual cells and the reduction of the protoplast volume was quantified (Zeiss AxioVision 4.7.1 software).

In this way, assuming a cylindrical shape, the protoplast volume at saturation (V_s_ in Eq. (1)) and after desiccation (V_d_ in Eq. (2)) was calculated by the following equations (for abbreviations, see Fig. 1):

(1) Vsμm3= CLD 22*π* CLL

(2) $$V_{\;d\left[\mu m^{3}\right]}\;={\left(\frac{PD_{d}}{2}\right)}^{2}\;*\pi *PL_{d}.$$

The RV_RED_ (%) of the PD_d_ related to the protoplast volume at the initial, saturated state was determined according to Eq. (3).

(3) RV RED %=Vs−VdVs×100.

RV_RED_ were then plotted against RH values of both studied species.

### Statistical evaluation

Significant differences between mean values of RV_RED_ [%] for each investigated genus (*K. crenulatum*, *Zygnema* sp.) were calculated with one‐way ANOVA followed by Games–Howell's *post hoc* test; *p* < 0.01) using SPSS software. Significant differences of RV_RED_ [%] between the two genera at a certain RH level were calculated by Student's *t*‐test (*p* < 0.05) by SPSS software.

## Results

### Test chamber

We first tested our newly constructed test chamber for functionality at different RH. The chamber was filled with the respective LiCl solutions of different concentrations or the saturated K_2_CO_3_ solution or with silica gel to generate different levels of RH (Table 2). At high levels of RH (>80%), the RH inside the test chamber reached the saturation point within 3–4 h (Fig. 3), over silica gel, the saturation point was achieved in less than 3 h (Fig. 3). The recorded RH differed only slightly between experiments (Table 2).

### Measurements of volume reduction

After transfer of algal suspensions into the test chamber, an initial phase was observed, where only culture medium evaporated and the cells did not show any change in volume or shape. Then, the desiccation process started, in which the algae lost water for about ∼15–20 min until they reached an equilibrium state. The image series in Figure 4 shows cell filaments of *K. crenulatum* (78.3% RH, 90–140 min after start of the experiment; Fig. 4A) and *Zygnema* sp. (56.4% RH, 155–190 min after start of experiment; Fig. 4B) during the desiccation phase.

**Figure 2:**
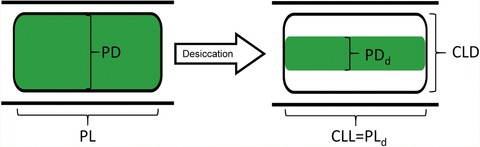
Schematic drawing of the change of protoplast shape during the desiccation process. The parameters, which were used for determining the protoplast volume before (volume in the saturated state, V_s_) and after (volume in the desiccated state, V_d_) desiccation, are depicted. Abbreviations: PD = protoplast diameter; PL = protoplast length; CLD = cell lumen diameter after desiccation; CLL = cell lumen length after desiccation; PD_d_ = protoplast diameter after desiccation; PL_d_ = protoplast length after desiccation.

The diameter of the protoplasts experienced a drastic reduction during the desiccation process, whereas the length of the protoplasts usually did not change and the protoplasts remained attached to the cross‐walls (Figs. 2 and 4A). Only in *Zygnema* sp., protoplasts occasionally were detached from the cross‐walls, which lead to shrinkage in length during the desiccation process (Fig. 4B). As in these cases, severe damage was expected; these cells were not included for volume calculations. In *Zygnema* sp., the strongest shrinkage of the protoplasts was observed in the central area and therefore a mean value was used to calculate the volume reduction (see Figs. 5A and B). In some cases, the PD_d_ even disintegrated into portions, resembling the chloroplast centres and pyrenoids (see Figs. 5C–E); such protoplasts were not used for measurements.

**Figure 3:**
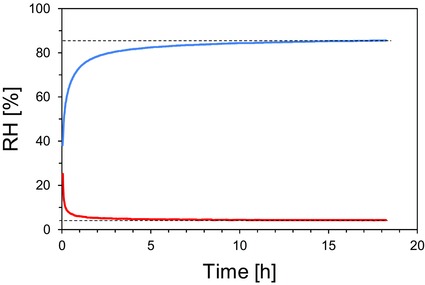
Exemplary presentation of time courses of the RH inside the test chamber using a 13 g/100 mL LiCl solution (blue line) and silica gel (red line). The target moisture content of 85.6% and 4.2%, respectively, is indicated by horizontal dotted lines.

**Figure 4:**
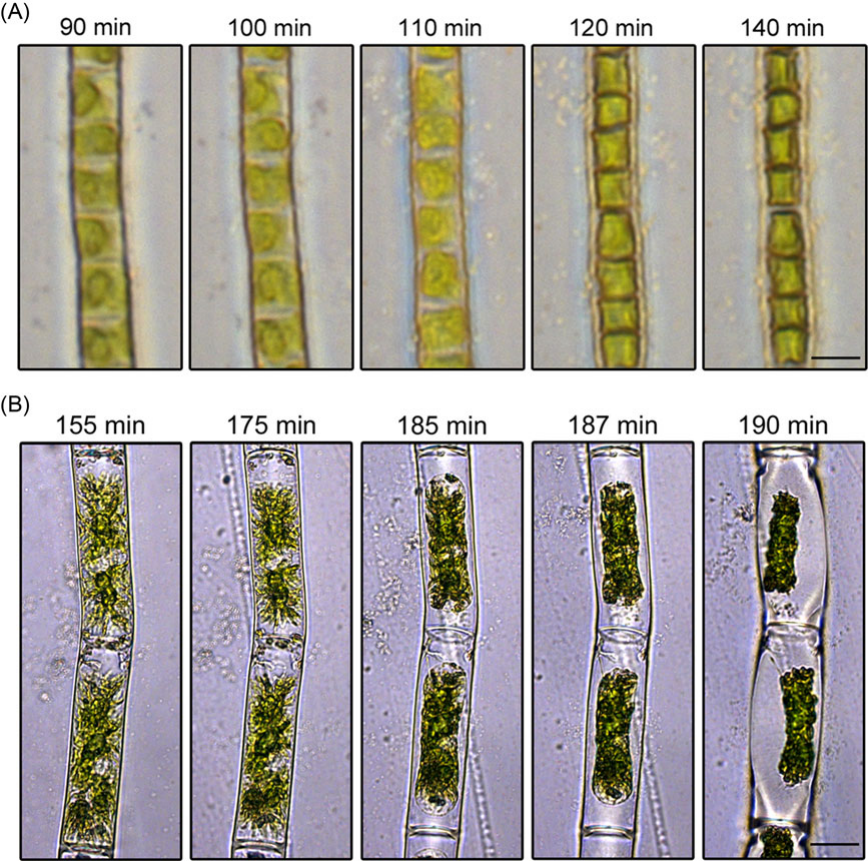
Light microscopic image series of the desiccation process in (A) *Klebsormidium crenulatum* at 78.3% RH, Scale bar 5 μm and (B) in *Zygnema* sp. at 56.4% RH, time in minutes (min) after the start of the desiccation experiment. Scale bar 20 μm.

**Figure 5:**
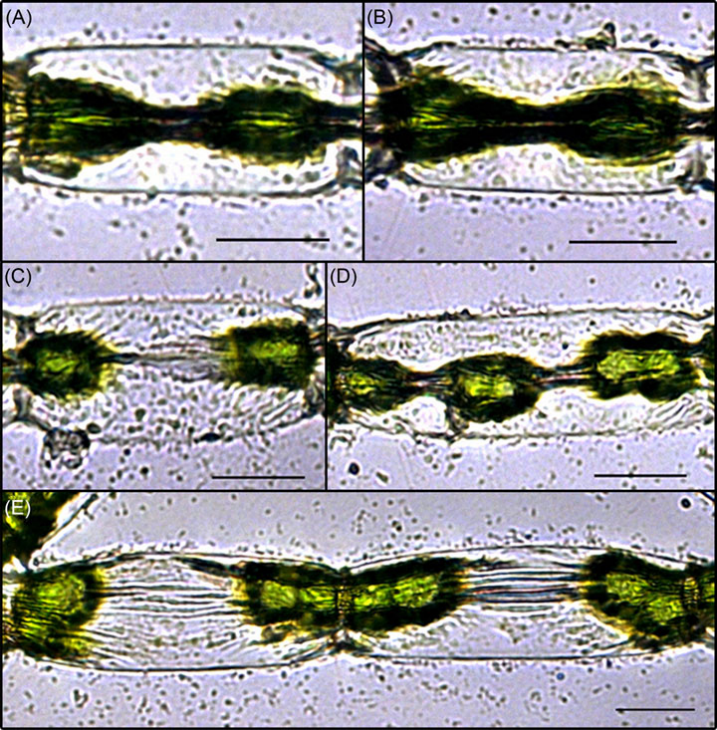
Light microscopic images of *Zygnema* sp. cells after 3 h 20 min desiccation at 56.4% RH. Images (A) and (B) represent examples of ‘hourglass’ shape. Images (C)–(E) show cases where the protoplast of *Zygnema* sp. disintegrated as an effect of the desiccation. Scale bars 20 μm.

**Figure 6:**
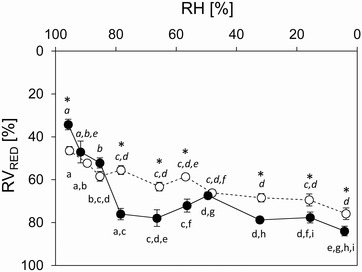
Relative volume reduction (RV_RED_ [%]; mean values ± SE) in *Klebsormidium crenulatum* (open symbols) and *Zygnema* sp. (solid symbols) determined at certain RH levels. Significant differences between mean values of RV_RED_ [%] for each investigated genus (*K. crenulatum*, *Zygnema* sp.) are indicated by different letters (normal font: *Zygnema* sp., italics: *K. crenulatum*; one‐way ANOVA followed by Games–Howell's *post hoc* test; *p* < 0.01). Significant differences of RV_RED_ [%] between the two genera at a certain RH level are indicated by asterisks (Student's *t*‐test; *p* < 0.05).

Calculations of the RV_RED_ were performed by measuring the protoplast diameter after a constant level of RH inside the test chamber was achieved and cells did not show further changes in protoplast volume or shape. In *K. crenulatum*, the RV_RED_ values (mean ± SE) ranged from 46.4 ± 1.9% at 95.4% RH to 75.9 ± 2.7% at 3.7% RH (Fig. 6). In *Zygnema* sp., the RV_RED_ values of 34.3 ± 2.4% at the highest RH was significantly higher (*p* < 0.01) when compared to *K. crenulatum*. At the lowest RH the value of 83.9 ± 2.2% was significantly (*p* < 0.05) lower in comparison to *K. crenulatum*. The development of volume reductions differed between the two genera. Although in *K. crenulatum* a drastic water loss was observed already at highest RH tested, in *Zygnema* sp. the initial water loss was less pronounced. However, in *Zygnema* sp. the water loss at RH values <∼80% was more drastic indicated by higher RV_RED_ values.

## Discussion

In this study, a new test chamber allowed the monitoring of desiccation effects at certain preset RH during microscopic observation. This device was used with two green algae, *K. crenulatum* and *Zygnema* sp., for which the RV_RED_ was experimentally determined. A distinct desiccation behaviour was found by quantitative analysis of the RV_RED_ at different RHs. The RV_RED_ values at the highest RH tested (∼95% RH) indicated a stronger water loss in *K. crenulatum*. By contrast, at lower RHs *Zygnema* sp. showed a stronger water loss. There are also qualitative differences in the shape of the retracted chloroplasts between the two studied green algal genera.

In general, these observations correlate well with physiological parameters, e.g. reduction of the effective quantum yield as a consequence of desiccation in *K. crenulatum* (Karsten *et al*., 2010) and *Zygnema* sp. (Herburger *et al*., 2015). Herburger and Holzinger (2015) investigated these two genera in a parallel setup and monitored the desiccation and recovery effects of individual filaments by imagining PAM. In both genera, desiccation at ambient air (RH ∼65%) leads to a rapid decrease of the effective quantum yield of photosystem II (YII). Upon rehydration for 180 min, the Y II is re‐established, in *Klebsormidum cenulatum* to nearly the initial value and in *Zygnema* sp. to only half of the initial value (Herburger & Holzinger, 2015). In this study, we found clear differences between the two examined genera concerning the range of the RV_RED_‐RH relationship. Most important, RV_RED_ values below ∼80% RH are considerably higher in *Zygnema* sp. than *K. crenulatum*. This means that the protoplasts in *Zygnema* sp. experience a clearly stronger reduction in volume under similar RH levels. This correlates well with the observations by Kaplan *et al*. (2012, 2013), and may be attributed to the higher vacuolization status of young *Zygnema* sp. protoplasts (Kaplan *et al*., 2013; Herburger & Holzinger, 2015). Moreover, this could also explain the weaker recovery of YII in *Zygnema* sp. (Herburger & Holzinger, 2015). In *Zygnema* sp., older cultures form preakinetes, which are in general more stress tolerant (e.g. Pichrtová *et al*., 2014), and likely have different osmotic potentials (e.g. Fuller, 2013; Kaplan *et al*., 2013). In this study, we used young cultures, to exclude this phenomenon. However, the measurement of the *Zygnema* sp. protoplasts was more difficult to perform due to the shape of the desiccated cytoplasm.

In both genera, only individual cells from solitary filaments were measured. Bundles of filaments showed a slower desiccation process and so a prolonged moistness was observed. This is a well‐described strategy for filamentous, soil crust inhabiting organisms to enhance the water holding capacities in nature (e.g. Holzinger & Karsten, 2014). In the experiment, however this leads to complications with the measurements (overlapping of filaments, protoplast shape) and was therefore avoided. Moreover, we used for the calculation only cells where the protoplast remained attached to the cross‐walls, assuring that the cells were not damaged too badly had the potential to recover (Herburger & Holzinger, 2015).

Summarizing the results of this study, here we introduce a functional test chamber suitable to follow the desiccation process during microscopic observation. We demonstrated that the test chamber could create and maintain a constant RH during the desiccation experiments. These conditions allowed to subsequently calculate the volume reduction of the test organism *in situ*. The more rapid water loss observed in *K. crenulatum* might in nature be compensated by their habitat in soil crusts, giving protection against desiccation (Karsten & Holzinger, 2014). By contrast, *Zygnema* sp. collected from the sandy litoral zone of a river (Herburger *et al*., 2015) might be naturally exposed more frequently to higher RHs, which they are capable to tolerate. The stronger water loss at lower RHs resulted in severe damage, and it has been shown that particularly young vegetative *Zygnema* sp. cells cannot recover from desiccation over silica gel, whereas they tolerate desiccation at ∼83% RH with good recovery success according to measurements of the effective quantum yield (Pichrtová *et al*., 2014). We are aware that comparing genera after investigating only one strain per genus might lead to misinterpretations; therefore, future studies should include more strains.

The chamber presented here will allow monitoring desiccation kinetics under defined RHs. Repeated desiccation and rehydration cycles could be created in order to study possible legacy effects. Future studies should elucidate which osmotic compounds are responsible for water holding capacities that enable aeroterrestrial green algae to withstand desiccation. Additionally, this chamber might also be valuable for further studies measuring the photosynthetic activity of single cells during desiccation, which is possible with new‐generation chlorophyll fluorimeters or the observation of other physiological responses employing fluorescent probes, e.g. H2DCF‐DA to detect the formation of reactive oxygen species during the desiccation process.

## References

[jmi12409-bib-0001] Beckett, R.P. (1995) Some aspects of the water relations of lichens from habitats of contrasting water status studied using thermocouple psychrometry. Ann. Bot. 76, 211–217.

[jmi12409-bib-0002] Beckett, R.P. (1997) Pressure–volume analysis of a range of poikilohydric plants implies the existence of negative turgor in vegetative cells. Ann. Bot. 79, 145–152.

[jmi12409-bib-0003] Bischoff, H.W. & Bold, H.C. (1963) Phycological studies IV. Some soil algae from enchanted rock & related algal species. Univ. of Texas Publ. 6318, 1–95.

[jmi12409-bib-0004] Delaux, P.‐M. , Nanda, A.K. , Mathé, C. , Sejalon‐Delmas, N. & Dunand, C. (2013) Molecular and biochemical aspects of plant terrestrialization. Persp. Plant Ecol. Evol. 14, 49–59.

[jmi12409-bib-0005] Delwiche, C.F. & Cooper, E.D. (2015) The evolutionary origin of a terrestrial flora. Curr. Biol. 25(19), R899–R910. 26439353 10.1016/j.cub.2015.08.029

[jmi12409-bib-0006] Fuller, C.L. (2013) Examining morphological and physiological changes in Zygnema irregulare during a desiccation and recovery period. Master's Thesis, California State University.

[jmi12409-bib-0007] Graham, L.E. , Arancibia‐Avila, P. , Taylor, W.A. , Strother, P.K. & Cook, M.E. (2012) Aeroterrestrial *Coleochaete* (Streptophyta, Coleochaetales) models early plant adaptation to land. Am. J. Bot. 99, 130–144. 22210844 10.3732/ajb.1100245

[jmi12409-bib-0008] Hájek, T. & Beckett, R.P. (2008) Effect of water content components on desiccation and recovery in *Sphagnum* mosses. Ann. Bot. 101, 165–173. 18024417 10.1093/aob/mcm287 PMC2701845

[jmi12409-bib-0009] Hay, F.R. , Adams, J. , Manger, K. & Probert, R. (2008) The use of non‐saturated lithium chloride solutions for experimental control of seed water content. Seed Sci. Technol. 36, 737–746.

[jmi12409-bib-0010] Herburger, K. & Holzinger, A. (2015) Localization and quantification of callose in the streptophyte green algae *Zygnema* and *Klebsormidium*: correlation with desiccation tolerance. Plant Cell Physiol. 56, 2259–2270. 26412780 10.1093/pcp/pcv139 PMC4650865

[jmi12409-bib-0011] Herburger, K. , Lewis, L.A. & Holzinger, A. (2015) Photosynthetic efficiency, desiccation tolerance and ultrastructure in two phylogenetically distinct strains of alpine *Zygnema* sp. (Zygnematophyceae, Streptophyta): role of pre‐akinete formation. Protoplasma 252, 571–589. 25269628 10.1007/s00709-014-0703-3 PMC4335129

[jmi12409-bib-0012] Holzinger, A. & Karsten, U. (2013) Desiccation stress and tolerance in green algae: consequences for ultrastructure, physiological and molecular mechanisms. Front. Plant. Sci. 4, 327. doi: 10.3389/fpls.2013.00327. 23986769 10.3389/fpls.2013.00327 PMC3749462

[jmi12409-bib-0013] Holzinger, A. , Kaplan, F. , Blaas, K. , Zechmann, B. , Komsic‐Buchmann, K. & Becker, B. (2014) Transcriptomics of desiccation tolerance in the streptophyte green alga *Klebsormidium* reveal a land plant‐like defense reaction. PLoS ONE 9, e110630. doi:10.1371/journal.pone.0110630 25340847 10.1371/journal.pone.0110630 PMC4207709

[jmi12409-bib-0014] Hori, K. , Maruyama, F. , Fujisawa, T. , *et al* (2014) *Klebsormidium flaccidum* genome reveals primary factors for plant terrestrial adaptation. Nat. Commun. 5, 3978. doi:10.1038/ncomms4978. 24865297 10.1038/ncomms4978 PMC4052687

[jmi12409-bib-0015] Kaplan, F. , Lewis, L.A. , Wastian, J. & Holzinger, A. (2012) Plasmolysis effects and osmotic potential of two phylogenetically distinct alpine strains of *Klebsormidium* (Streptophyta). Protoplasma 249, 789–804. 21979310 10.1007/s00709-011-0324-z

[jmi12409-bib-0016] Kaplan, F. , Lewis, L.A. , Herburger, K. & Holzinger, A. (2013) Osmotic stress in Arctic and Antarctic strains of the green alga *Zygnema* (Zygnematales, Streptophyta): effects on photosynthesis and ultrastructure. Micron 44, 317–330. 22959821 10.1016/j.micron.2012.08.004 PMC3523258

[jmi12409-bib-0017] Karsten, U. & Holzinger, A. (2014) Green algae in alpine biological soil crust communities: acclimation strategies against ultraviolet radiation and dehydration. Biodivers. Conserv. 23, 1845–1858. 24954980 10.1007/s10531-014-0653-2 PMC4058318

[jmi12409-bib-0018] Karsten, U. , Lütz, C. & Holzinger, A. (2010) Ecophysiological performance of the aeroterrestrial green alga *Klebsormidium crenulatum* (Charophyceae, Streptophyta) isolated from an alpine soil crust with an emphasis on desiccation stress. J. Phycol. 46, 1187–1197. 10.1111/j.1529-8817.2011.00980.x 27021989

[jmi12409-bib-0019] Leliaert, F. , Smith, D.R. , Moreau, H. , Herron, M.D. , Verbruggen, H. , Delwiche, C.F. & De Clerck, O. (2012) Phylogeny and molecular evolution of the green algae. Crit. Rev. Plant Sci. 31, 1–46.

[jmi12409-bib-0020] Lokhorst, G.M. & Star, W. (1985) Ultrastructure of mitosis and cytokinesis in *Klebsormidium mucosum* nov. comb., formerly *Ulothrix verrucosa* (Chlorophyta). J. Phycol. 21, 466–476.

[jmi12409-bib-0021] Mikhailyuk, T. , Holzinger, A. , Massalski, A. & Karsten, U. (2014) Morphological and ultrastructural aspects of *Interfilum* and *Klebsormidium* (Klebsormidiales, Streptophyta) with special reference to cell division and thallus formation. Eur. J. Phycol. 49, 395–412. 26504365 10.1080/09670262.2014.949308 PMC4618308

[jmi12409-bib-0022] Mikhailyuk, T. , Glaser, K. , Holzinger, A. & Karsten, U. (2015) Biodiversity of *Klebsormidium* (Streptophyta) from alpine biological soil crusts (Alps, Tyrol, Austria and Italy), J. Phycol. 51, 750–767. 26504252 10.1111/jpy.12316 PMC4618304

[jmi12409-bib-0023] Nardini, A. , Marchetto, A. & Tretiach, M. (2013) Water relation parameters of six *Peltigera* species correlate with their habitat preferences. Fungal Ecol. 6, 397–407.

[jmi12409-bib-0024] Pichrtová, M. , Kulichová, J. & Holzinger, A. (2014) Nitrogen limitation and slow drying induce desiccation tolerance in conjugating green algae (Zygnematophyceae) from polar habitats. PLOS one 9(11), e113137. 25398135 10.1371/journal.pone.0113137 PMC4232603

[jmi12409-bib-0025] Proctor, M.C. , Nagy, Z. , Csintalan, Z. & Takács, Z. (1998) Water‐content components in bryophytes: analysis of pressure‐volume relationships. J. Exp. Bot. 49, 1845–1854.

[jmi12409-bib-0026] Rindi, F. , Mikhailyuk, T.I. , Sluiman, H.J. , Friedl, T. & López‐Bautista, J.M. (2011) Phylogenetic relationships in *Interfilum* and *Klebsormidium* (Klebsormidiophyceae, Streptophyta). Mol. Phylogenet. Evol. 58(2), 218–231. 21145975 10.1016/j.ympev.2010.11.030

[jmi12409-bib-0027] Sørensen, I. , Pettolino, F.A. , Bacic, A. , Ralph, J. , Lu, F. & O'Neill, M.A. , et al. (2011) The charophycean green algae provide insights into the early origins of plant cell walls. Plant J. 68, 201–221. 21707800 10.1111/j.1365-313X.2011.04686.x

[jmi12409-bib-0028] Starr, R.C. & Zeikus, J.A. (1993) UTEX—the culture collection of algae at the University of Texas at Austin 1993 list of cultures. J. Phycol. 29(s2), 1–106.

[jmi12409-bib-0029] Timme, R.E. , Bachvaroff, T.R. & Delwiche, C.F. (2012) Broad phylogenomic sampling and the sister lineage of land plants. PLoS One 7, e29696. 22253761 10.1371/journal.pone.0029696 PMC3258253

[jmi12409-bib-0030] Tuba, Z. , Lichtenthaler, H.K. , Csintalan, Z. , Nagy, Z. & Szente, K. (1994) Reconstitution of chlorophylls and photosynthetic CO_2_ assimilation upon rehydration of the desiccated poikilochlorophyllous plant *Xerophyta scabrida* (Pax) Th. Dur. et Schinz. Planta 192, 414–420.

[jmi12409-bib-0031] Walter, H. & Stadelmann, E. (1974) A new approach to the water relations of desert plants. Desert Biol. 2, 213–310.

[jmi12409-bib-0033] Wodniok, S. , Brinkmann, H. , Glöckner, G. , Heidel, A.J. , Philippe, H. , Melkonian, M. & Becker, B. (2011).Origin of land plants: do conjugating green algae hold the key? BMC Evol. Biol. 11, 104. doi: 10.1186/1471-2148-11-104. 21501468 10.1186/1471-2148-11-104 PMC3088898

